# Supplementary material for: Virtual body ownership and its consequences for implicit racial bias are dependent on social context
Source: R Soc Open Sci. 2020 Dec 9;7(12):201848. doi: 10.1098/rsos.201848 (PMC7813259; doi:10.1098/rsos.201848)
Supplement: Supplementary Information [file rsos201848supp1.pdf]

SUPPLEMENTARY INFORMATION: Virtual body ownership and its consequences for implicit racial bias are dependent on social context

Domna Banakou[1,2], Alejandro Beacco[1], Solène Neyret[1], Marta Blasco-Oliver[1], Sofia Seinfeld[3+], Mel Slater[1,2]*

[1] Event Lab, Department of Clinical Psychology and Psychobiology, University of Barcelona, Barcelona, Spain
[2] Institute of Neurosciences of the University of Barcelona, Barcelona, Spain
[3] IDIBAPS, Barcelona, Spain

* Corresponding author: melslater@ub.edu

Supplementary Information

S1 Virtual Agents and Crowd Behavior 1

S2 Implicit Association Test (IAT) 2

S3 Attitudes Towards Blacks (ATB) Scale 2

S4 Evaluation of the crowd 2

Supplementary Tables 4

Supplementary Figures 6

Supplementary Video 11

Supplementary References 11

S1 Virtual Agents and Crowd Behaviour

Although the crowd consisted of 80 virtual characters in fact, for rendering efficiency, there were only 10 different types of virtual human agent (5 male and 5 female) with random sets of colours within a specific palette (avoiding strange combinations). We designed the palette to have the representative features of Spanish people (e.g. hair colour more likely to be brunette than blonde, skin perceived as white). Every agent had its own 3D high level-of-detail polygonal mesh and 2 low level-of-detail polygonal meshes to use from far distance using a Level of Detail technique [1]. Facial expressions were achieved using blendshapes on the high polygonal meshes. Animation clips were retrieved from Mixamo and synthesized using blendtrees for locomotion [2]. The simulation used the Unity navigation system, and the Entity Component System (ECS) for improved performance [Unity. Performance by default. https://unity.com/dots (2019)]. In an ECS every agent is an entity with a set of components (navigation, behaviour, animation, etc.), and we program component systems that

+ Current address: Image Processing and Multimedia Technology Center, Polytechnic University of Catalonia, Barcelona, Spain.

execute the code for a group of components. For example, we have a system that computes the navigation of all entities with the navigation component. The entities only have the data needed to work, in the case of the navigation it would be the position and orientation in the world. The navigation system retrieves all the entities that have a navigation system, loops through all of them, and moves them properly.

S2 Implicit Association Test (IAT)

Implicit racial bias was measured with a racial IAT [3], which was administered to participants twice: between 6 and 9 days before their virtual exposure (*preIAT*) and immediately after (*postIAT*), following earlier examples (2, 3). The IAT was completed on the same desktop screen both times. The test followed the standard IAT procedures (4) and had been previously used in the studies of [4, 5]. Participants were required to rapidly categorize faces (Black or White) and words (positive or negative) into groups. Implicit bias is calculated from the differences in accuracy and speed between categorizations (e.g., White people's faces, positive words and Black people's faces, negative words compared to the opposite groups). Higher IAT scores are interpreted as greater implicit racial bias, as this signifies longer reaction times and greater inaccuracies in categorizing Black people's faces with positive words, and White faces with negative words. Here the response variable of interest was *postIAT* with *preIAT* as a covariate to examine whether the VR exposure led to any changes in implicit bias against Black people. It has been shown that IAT scores tend to show slightly stronger associations corresponding to the pairings of the combined block that is completed first [6], the order of the combined blocks was counterbalanced among participants [7].

S3 Attitudes Towards Blacks (ATB) Scale

Explicit racial bias was measured with the Attitudes Towards Black (ATB) scale, which is designed to address negative behaviour (discomfort in close interactions is measured too) towards Black people (Cronbach's alpha .88, social desirability rating 1.75) [8]. The scale consists of 20 items, which are rated on a 1-5 Likert scale from "Strongly Agree" to "Strongly Disagree", with 10 reversed scored items. Examples include statements such as "*If I had a chance to introduce black visitors to my friends and neighbours, I would be pleased to do so*", "*Black and white people are inherently equal*", or "*Generally, blacks are not as smart as whites*" and "*I enjoy a funny racial joke, even if some people might find it offensive*" etc. For the present study one statement was removed from the questionnaire as it did not apply to Spain ("*I favour open housing laws that allow more racial integration of neighbourhoods*"). The ATB questionnaire was administered between 6 and 9 days before participants' virtual exposure (*preATB*) and again immediately after their exposure (*postATB*). To reduce possible response bias, the ATB items were presented in different order pre- and post-exposure. Higher ATB scores (with a maximum of 100 points, here 95 point because of the excluded item) are interpreted as less explicit racial bias. The response variable of interest was *postATB* with *preATB* as a covariate to examine whether the VR exposure led to any changes in explicit bias against Black people.

S4 Evaluation of the crowd

A questionnaire to assess the impact of the crowd on affect is shown in Table S2. Figure S1 shows the box plots of the scores from these questions, suggesting that the Negative crowd elicited negative participant responses compared to the Neutral and Positive conditions.

Cronbach's alpha for the whole set of 10 questions is 0.92, which is high indicating test score reliability. In order to present these results in a more succinct form a principle components factor

analysis with varimax rotation was carried out on all the scores of Table S2. Two factors were retained, the first accounting for 37% of the variance and the second a further 32%. The factor loadings are plotted against one another in Figure S2.

From Figure S2, the group of variables *friendliness*, *group*, *gazepositive* and *distancepositive* have negative loadings on both factors, the remaining variables positive factor loadings.

Considering the factor loadings > 0.5, the first factor is associated with:

- The crowd avoiding (*avoidance*)
- Feeling of the crowd keeping a distance (*distance*)
- The crowd rejecting (*rejection*).

The second factor is associated with:

- The crowd looking towards the participant (*gaze*)
- This resulted in negative feelings (*gazenegative*)
- The crowd were judging (*judgement*)
- Feeling nervous about the distance kept by the crowd (*distancenegative*)
- The crowd rejecting (*rejection*)

The two factors are different expressions of *negative* feelings. The first (*yaffect1*) is more concerned with the avoidance and effects of distance and the second (*yaffect2*) more concerned with the effects of gaze. Positive values here indicate greater negative affect.

Regression scores were generated from the two factors. Bar charts showing the means and standard errors of the two factors by condition are shown in Figure S3. For *yaffect1* (avoidance) there is greatest negative affect for the Negative crowd condition, and least negative affect for the Positive crowd, with the Neutral condition between these two. For *yaffect2* (gaze) the greatest difference is between the Negative and Neutral conditions. In the Negative condition the crowd engaged in negative gaze behaviour (e.g., looking at the participant with negative facial expressions) but in the Neutral condition the crowd members did not look towards the participant (except by chance). In the Positive condition, crowd members would sometimes acknowledge the participant with pleasant facial expressions.

A Bayesian ANOVA was carried out on the two scores *yaffect1* and *yaffect2*. Weakly informative priors were used for the means, normal distributions with mean 0 and standard deviation 10. The prior standard deviations were given Gamma distributions with shape 2 and rate 0.1 (as in the main paper). The posterior distributions are shown in Figure S4. These follow the same pattern as the bar charts (Figure S3). The main effect is due to Crowd rather than BodyType. For *yaffect1*, the posterior probability that the Negative condition is greater than the Neutral is 0.956, and the Neutral greater than the Positive is 0.952. The probability that the Negative condition is greater than both of the others is 0.956. For *yaffect2* the probability that the Negative condition is greater than the Neutral is 1.000. The probability that the Negative condition is greater than the positive is 0.85, as is the probability that the Negative condition mean is greater than both of the other two.

Supplementary Tables

Table S1 Experimental design and characteristics of the sample by condition. VR, and hours per week playing video games (1 = 0, 2 = “<1,” 3 = “1– 3,” ... ,6 = “7–9,” 7 = “>9”). Codes refer to a 1-7 Likert scale. For previous VR experience and hours spent playing video games, 1 means the least and 7 the most. Self-Esteem scores refer to Rosenberg’s scale, with higher scores indicating higher self-esteem.

	**BodyType**	
**Crowd**	**Black**	**White**
**Positive**	n = 15	n = 15
Mean ± SD Age	23.3 ± 4.0	20.3 ± 3.0
Median Code Previous VR Experience (IQR)	3(3)	3(2)
Median Code Games (IQR)	2(1)	1(0)
Mean ± SD Self-Esteem	31.5 ± 5.0	31.0 ± 4.1
**Negative**	n = 15	n = 15
Mean ± SD Age	21.2 ± 2.3	21.7 ± 3.2
Median Code Previous VR Experience (IQR)	2(3)	2(1)
Median Code Games (IQR)	1(0)	1(0)
Mean ± SD Self-Esteem	30.1 ± 4.3	32.4 ± 4.0
**Neutral**	n = 16	n = 16
Mean ± SD Age	21.9 ± 3.4	21.9 ± 2.4
Median Code Previous VR Experience (IQR)	1.5 (2.5)	1.5(2)
Median Code Games (IQR)	1(0)	1(0)
Mean ± SD Self-Esteem	30.0 ± 4.0	33.0 ± 5.0

Table S2 Post-exposure questionnaire on evaluation of the virtual crowd. The scores ranged from -3 (strongly disagree) to +3 (strongly agree).

Variable name	Statement
group	"I identified myself with the people of the crowd and I felt I was one more of them"
gaze	"I felt that people of the crowd were looking at me…"
gazenegative	"…and that situation made me feel nervous and/or uncomfortable"
gazepositive	"...and that situation made me feel untroubled and/or comfortable"
distance	"I felt that people of the crowd were keeping distance from me…"
distancenegative	"...and that situation made me feel nervous and/or uncomfortable"
distancepositive	"...and that situation made me feel untroubled and/or comfortable"
avoidance	"I felt that people of the crowd were avoiding me"
judgement	"I felt I was being judged by the people of the crowd"
rejection	"I felt I was being rejected by the people of the crowd"
friendliness	"The people in the crowd looked friendly"

Supplementary Figures

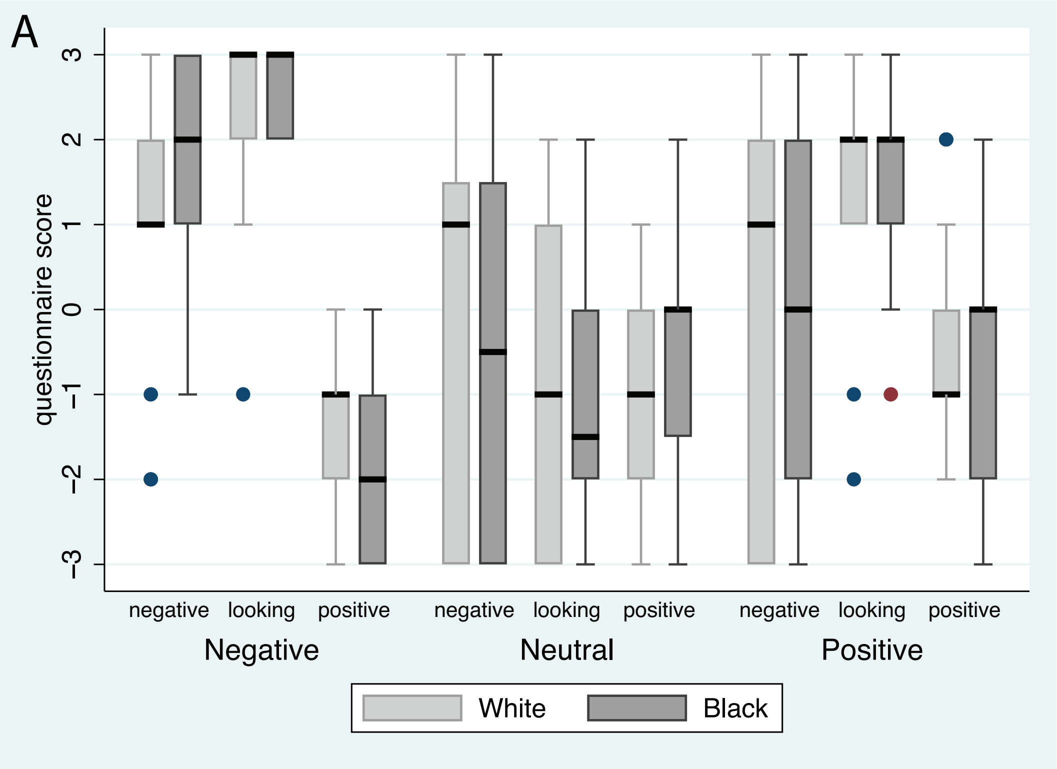

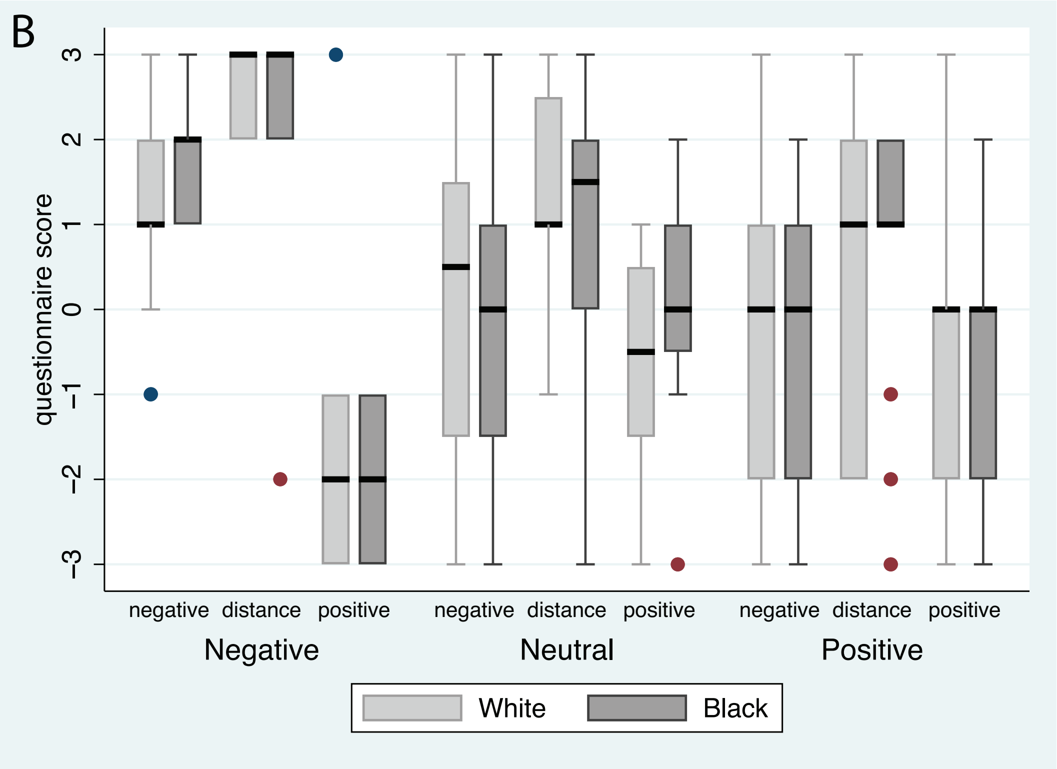

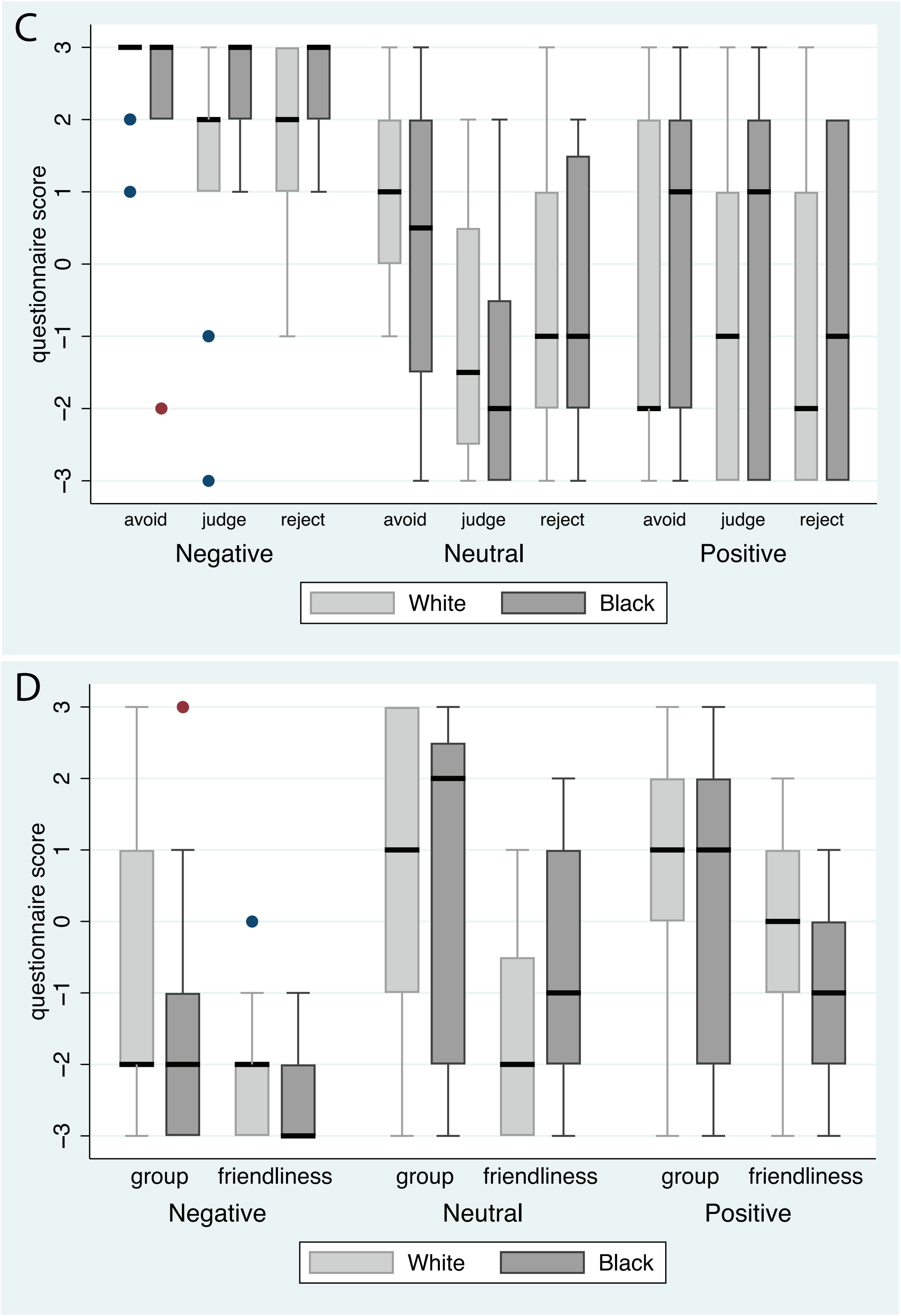

Figure S1 - Box plots for questionnaire responses about general evaluation of the crowd (Table S2) by condition. (A) Evaluation of gaze where negative, looking and positive refer to gazenegative, gaze and gazepositive. (B) Evaluation of distance where negative, distance, positive refer to distancenegative, distance and distance positive. (C) Overall negative evaluation of the crowd where avoid, judge, reject refer to avoidance, judgement and rejection. (D) Overall positive evaluation of the crowd with group and friendliness.

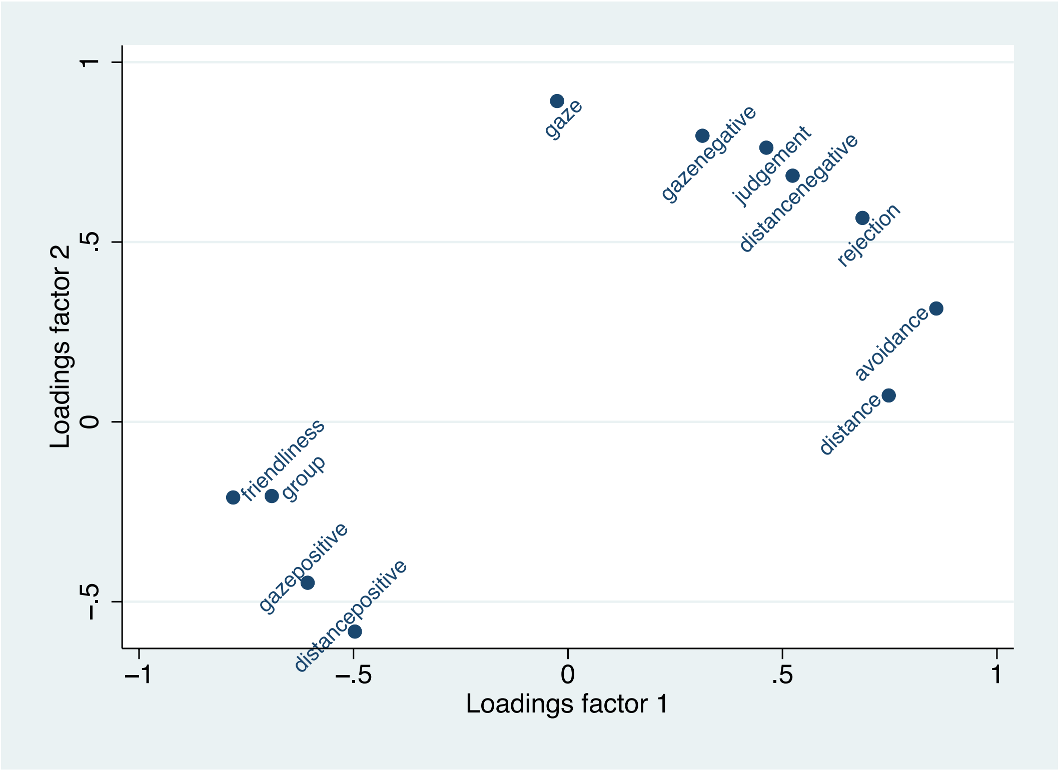

Figure S2 – Scatter diagram of factor loadings for factor 2 by factor 1 for the questions of Table S2.

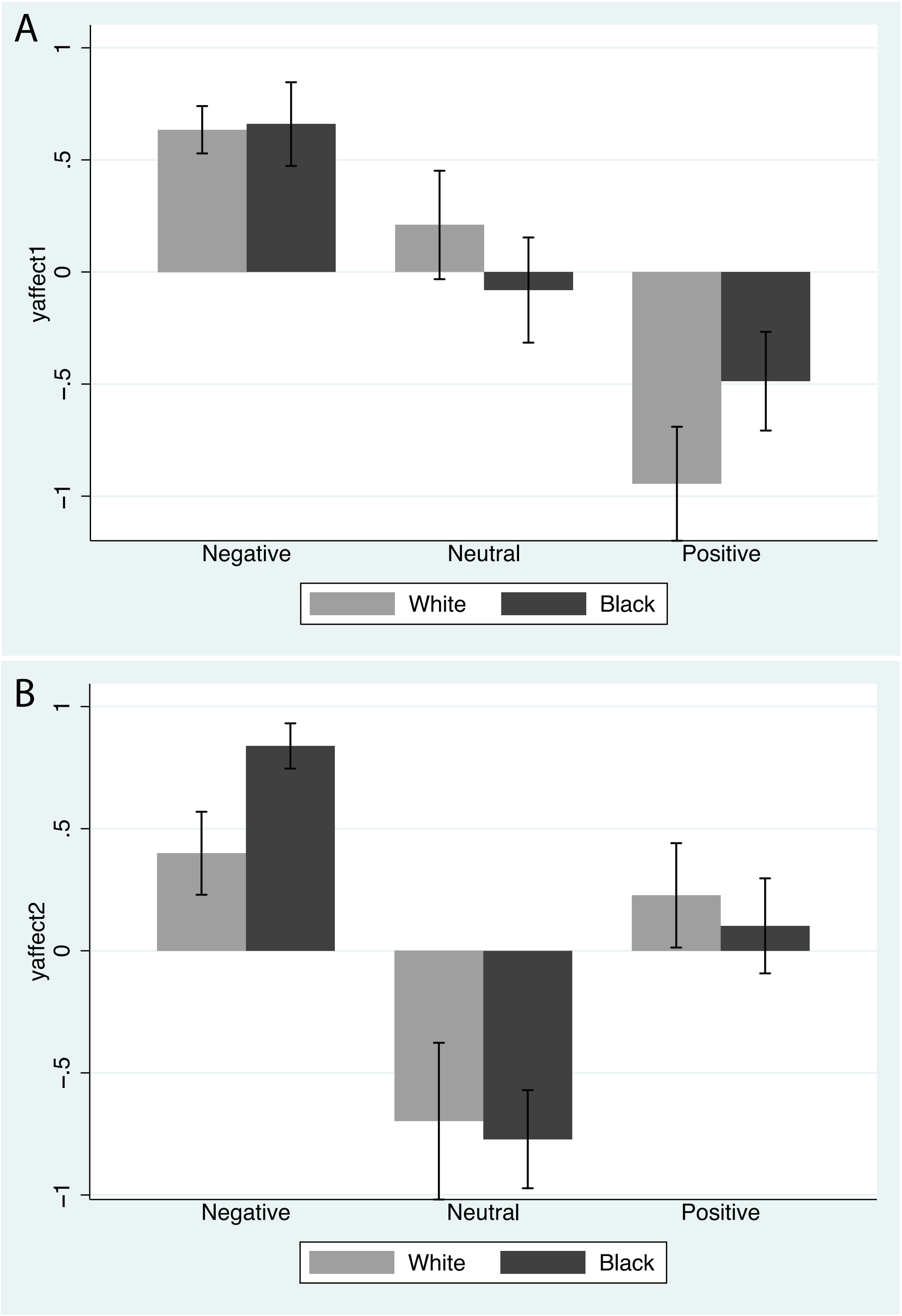

Figure S3 – Bar charts showing means and standard errors of the factor scoring variables (A) yaffect1 (avoidance, distance) (B) yaffect2 (gaze).

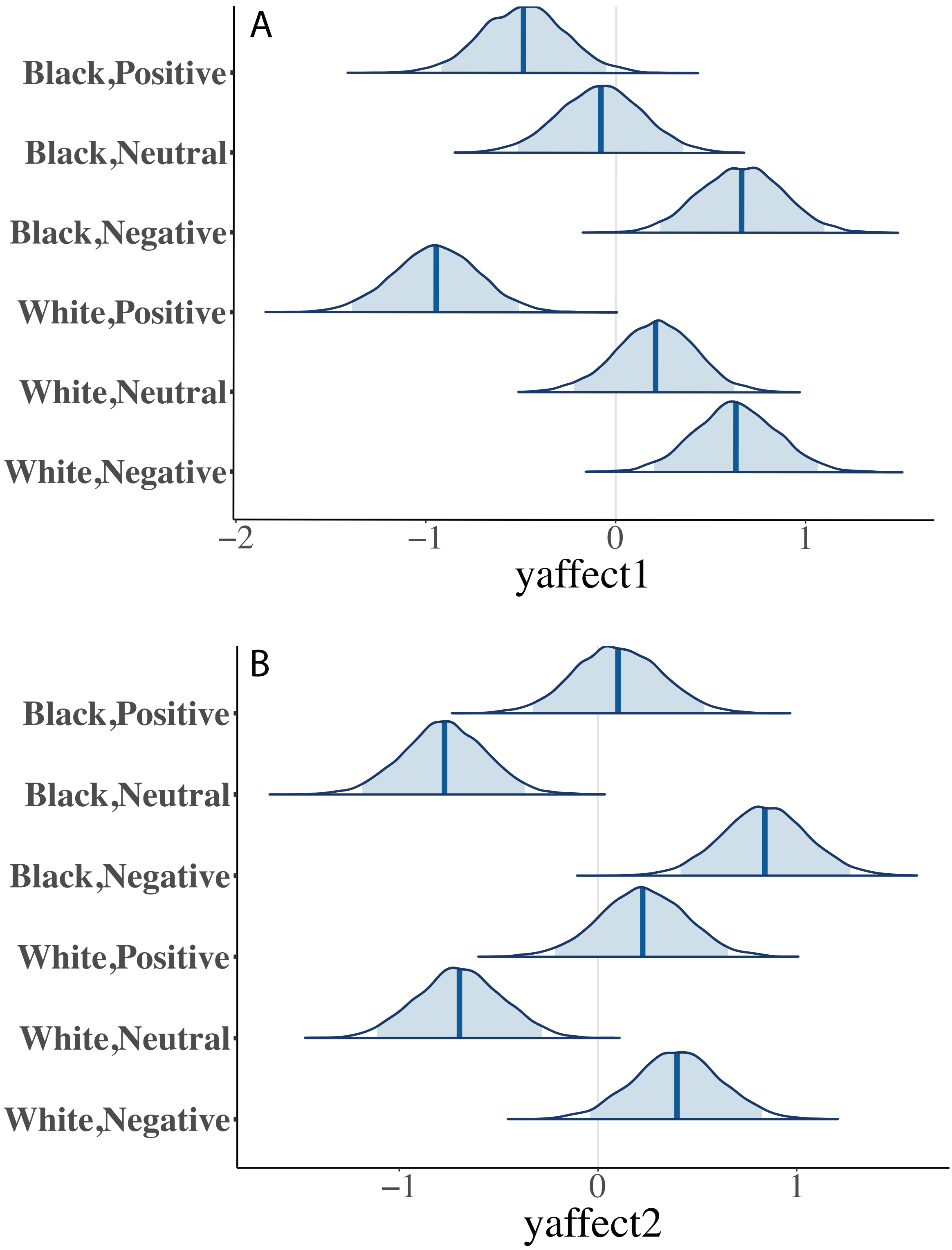

Figure S4 – Posterior distributions of the means for (A) yaffect1 (avoidance, distance) and (B) yaffect2 (gaze).

Supplementary Video

S1 Video – This video illustrates all the main features of the experiment – the embodiment period, the body types, and the negative, neutral and positive crowds.

Supplementary References

1 Luebke, D., Reddy, M., Jonathan, Cohen, D., Varshney, A., Watson, B., Huebner, R. 2002 *Level of Detail for 3D Graphics*. Morgan Kaufmann.
2 Feng, A., Huang, Y., Kallmann, M., Shapiro, A. Year An analysis of motion blending techniques. International Conference on Motion in Games; 2012: Springer; 2012. p. 232-243.
3 Greenwald, A. G., McGhee, D. E., Schwartz, J. L. K. 1998 Measuring individual differences in implicit cognition: The implicit association test. *Journal of Personality and Social Psychology*. **74**, 1464.
4 Peck, T. C., Seinfeld, S., Aglioti, S. M., Slater, M. 2013 Putting yourself in the skin of a black avatar reduces implicit racial bias. *Consciousness and cognition*. **22**, 779-787. (10.1016/j.concog.2013.04.016)
5 Banakou, D., PD, H., Slater, M. 2016 Virtual Embodiment of White People in a Black Virtual Body Leads to a Sustained Reduction in their Implicit Racial Bias. *Frontiers in Human Neuroscience*. **10:601**, (10.3389/fnhum.2016.00601)
6 Nosek, B. A., Greenwald, A. G., Banaji, M. R. 2005 Understanding and using the Implicit Association Test: II. Method variables and construct validity. *Personality and Social Psychology Bulletin*. **31**, 166-180.
7 Nosek, B. A., Greenwald, A. G., Banaji, M. R. 2007 The Implicit Association Test at age 7: A methodological and conceptual review. *Automatic processes in social thinking and behavior*. 265-292.
8 Brigham, J. C. 1993 College students' racial attitudes. *Journal of Applied Social Psychology*. **23**, 1933-1967.
